# Presynaptic hyperpolarization induces a fast analogue modulation of spike-evoked transmission mediated by axonal sodium channels

**DOI:** 10.1038/ncomms10163

**Published:** 2015-12-10

**Authors:** Sylvain Rama, Mickaël Zbili, Andrzej Bialowas, Laure Fronzaroli-Molinieres, Norbert Ankri, Edmond Carlier, Vincenzo Marra, Dominique Debanne

**Affiliations:** 1 INSERM, UMR_S 1072, Faculté de Médecine Secteur Nord, Boulevard Pierre Dramard, Marseille 13344, France; 2 UNIS, Faculté de Médecine Secteur Nord, Boulevard Pierre Dramard, Marseille 13344, France; 3 Aix-Marseille University, 13015 Marseille, France

## Abstract

In the mammalian brain, synaptic transmission usually depends on presynaptic action potentials (APs) in an all-or-none (or digital) manner. Recent studies suggest, however, that subthreshold depolarization in the presynaptic cell facilitates spike-evoked transmission, thus creating an analogue modulation of a digital process (or analogue–digital (AD) modulation). At most synapses, this process is slow and not ideally suited for the fast dynamics of neural networks. We show here that transmission at CA3–CA3 and L5–L5 synapses can be enhanced by brief presynaptic hyperpolarization such as an inhibitory postsynaptic potential (IPSP). Using dual soma–axon patch recordings and live imaging, we find that this hyperpolarization-induced AD facilitation (h-ADF) is due to the recovery from inactivation of Nav channels controlling AP amplitude in the axon. Incorporated in a network model, h-ADF promotes both pyramidal cell synchrony and gamma oscillations. In conclusion, cortical excitatory synapses in local circuits display hyperpolarization-induced facilitation of spike-evoked synaptic transmission that promotes network synchrony.

## Abstract (web summary)

'Digital' spike-evoked transmission can be facilitated by slow subthreshold 'analogue' depolarisation of the presynaptic neuron. Here, the authors identify a novel, rapid form of digital-analogue facilitation in mammalian neurons whereby presynaptic hyperpolarisation enables de-inactivation of axonal Nav channels.

## Graphical abstract

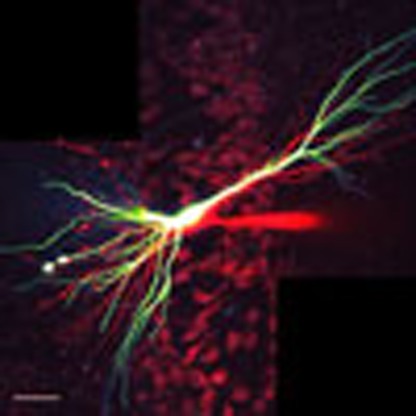

Neuronal information in the mammalian brain is generally transmitted through spike-evoked packets of neurotransmitter in a digital mode of signalling. It has been recently shown, however, that subthreshold analogue activity in the presynaptic element might modulate this digital signalling, leading to the emergence of the concept of a hybrid analogue–digital (AD) facilitation of synaptic transmission. Initially described in invertebrates^1^,^2^,^3^,^4^, this AD facilitation has been reported in many mammalian synapses including cortical^5^,^6^,^7^, cerebellar^8^,^9^ and hippocampal synapses^10^,^11^,^12^,^13^. This AD facilitation is induced by depolarization of the presynaptic neuron and is mediated by an elevation of glutamate release^6^,^9^,^14^. There are two independent mechanisms accounting for AD facilitation today^15^: (i) inactivation of D-type K^+^ channels^6^,^13^,^16^,^17^ and (ii) elevation in basal Ca^2+^ concentration mediated by the opening of P/Q-type voltage-gated Ca^2+^ channels^8^,^9^,^14^. Both forms of AD facilitation are slow processes because the underlying biophysical mechanism (inactivation of the D-type current or accumulation of Ca^2+^ in the axon) requires presynaptic voltage shifts lasting several seconds. Thus, these forms of AD modulation are not well suited for the fast network dynamics.

Analogue modulation by resting membrane potential is not limited to voltage-gated K^+^ and Ca^2+^ channels. In fact, a large fraction of the axonal voltage-gated Na^+^ (Nav) current is inactivated at the resting membrane potential^18^,^19^,^20^. The physiological consequence of Nav channel inactivation on axonal spike amplitude and synaptic transmission has not been clearly defined.

We show here that fast physiological hyperpolarization before the presynaptic action potential (AP) facilitates synaptic transmission in hippocampal and neocortical synaptic circuits. This hyperpolarization-induced AD facilitation (h-ADF) is due to the recovery of Nav channels from inactivation, thus enhancing the spike amplitude in the axon. Axonal recordings from L5 pyramidal neurons indeed revealed that hyperpolarization of the cell body specifically increased the spike amplitude in the axon but not in the cell body. The presence of h-ADF in different areas of the brain indicates that this form of plasticity can be generalized to a wide range of cortical excitatory synapses. A major physiological interest of this form of AD facilitation is its fast kinetics. h-ADF produced by inhibitory postsynaptic potential (IPSP)-spike sequences not only facilitates synaptic strength across pyramidal cells but also enhances synchrony and gamma oscillations in a network model. Thus, interneurons paradoxically enhance excitatory transmission between pyramidal neurons and subsequently structure network dynamics.

## Results

### Hyperpolarization-induced AD facilitation

**Figure 1:**
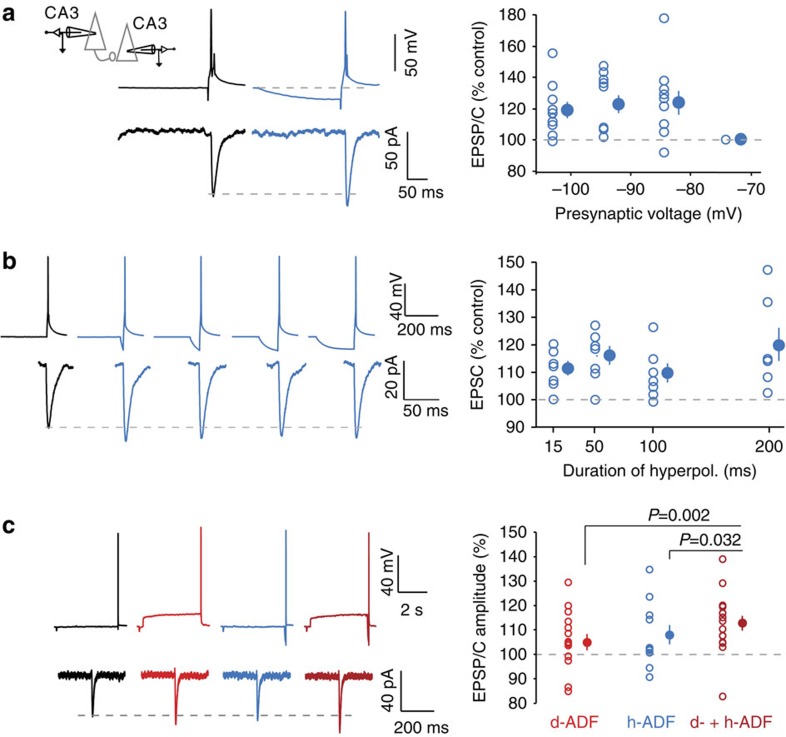
Synaptic facilitation induced by transient hyperpolarization (h-ADF) in CA3 neurons. (**a**) Facilitation of synaptic transmission at CA3–CA3 connections by a hyperpolarizing pre-pulse (200 ms duration). Left, schematic of the recording configuration. Middle, example of facilitation produced by the presynaptic hyperpolarizing pulse (10 traces were averaged). Right, summary of facilitation induced by presynaptic hyperpolarization of increasing amplitude. Note that no further facilitation was induced when the magnitude of the hyperpolarizing pre-pulse was increased. (**b**) h-ADF can be induced by brief presynaptic hyperpolarization. Left, examples of recording from a pair of connected CA3 pyramidal neurons with no hyperpolarization and 15, 50, 100 and 200 ms of hyperpolarization to −93 mV before the spike. Right, summary of facilitation induced by 15, 50, 100 and 200 ms (all Wilcoxon test, *P*<0.05, *n*=7). (**c**) d- and h-ADF are coexpressed at CA3–CA3 connections. Left, representative example. Top traces, membrane potential of the presynaptic neuron in control (black), during d-ADF (red), during h-ADF (blue) and when d- and h-ADF are combined (dark red). Bottom traces, postsynaptic responses in each case averaged over 10 trials. Right, group data (Mann–Whitney test, *n*=16, for d-ADF, 11 for h-ADF and 16 for d- and h-ADF). Note the stepwise increase in transmission when d- and h-ADF are combined.

We first measured the incidence of brief hyperpolarization of the presynaptic cell on synaptic transmission. Pairs of monosynaptically connected CA3 neurons were recorded in organotypic cultures of the rat hippocampus after 8–10 days *in vitro* (DIV)^21^. A 200-ms hyperpolarizing pre-pulse delivered before the presynaptic spike was found to increase synaptic strength by ∼20% (Fig. 1a). This increase was observed when measuring either amplitude or charge of the postsynaptic response (Supplementary Fig. 1). In these experiments, the presynaptic resting potential was −74±3 mV (*n*=10). The h-ADF was comparable when the presynaptic hyperpolarization amounted to −84 or −102 mV (respectively, 124±8% versus 119±5%, *n*=10; Wilcoxon test *P*>0.1), suggesting that a presynaptic hyperpolarization of ∼10 mV is sufficient to obtain saturating h-ADF. h-ADF was associated with a reduced paired-pulse ratio (PPR, from 99±7 to 88±5%, *n*=12; Wilcoxon test *P*<0.05; Supplementary Fig. 1), indicating that it results from a presynaptic increase in glutamate release.

A 200-ms-long hyperpolarization is unlikely to occur in a physiological context. Therefore, we investigated the time course of h-ADF for shorter hyperpolarizations (15, 50, 100 and 200 ms). h-ADF was observed for all the durations of hyperpolarization tested (15 ms: 111±3%, 50 ms: 116±4%, 100 ms: 109±4%, 200 ms: 120±6% Wilcoxon, *P*<0.05 for all durations, *n*=7, Fig. 1b). According to this result, h-ADF is likely to be induced by physiological hyperpolarization.

CA3 pyramidal neurons express depolarization-induced AD facilitation (d-ADF) that results from the slow inactivation of Kv1.1 channels (time constant: 3.3 s)^13^. We thus examined whether both d- and h-ADF were expressed at the same CA3–CA3 connections. Presynaptic APs were triggered alternatively from resting membrane potential (−78 mV control), after a long subthreshold depolarization (10 s, −62.6 mV, d-ADF), after a brief hyperpolarization (200 ms, −96.1 mV, h-ADF) or after the combination of a long depolarization and a brief hyperpolarization (d- and h-ADF; Fig. 1c, left). In fact, the combination of the two forms of ADF produced, in the same connections, a greater facilitation (113±3%, *n*=16; Fig. 1c) than that produced separately by each protocol (d-ADF alone: 105±3%, *n*=16, h-ADF alone: 108±4%, *n*=11; Fig. 1c). Notably, averaged h- and d-ADF were found to summate linearly, suggesting two independent molecular mechanisms. Moreover, d- and h-ADF measured in the same pairs were positively correlated (Supplementary Fig. 1), suggesting that some synaptic connections are more susceptible to AD facilitation, probably because analogue signal propagation along the axon depends on the distance between the soma and the presynaptic terminals. These data demonstrate that h- and d-ADF coexist in CA3 pyramidal neurons and that the underlying mechanisms are likely to be independent.

h-ADF was observed in young CA3 neurons (DIV8–10 prepared from P5–P7 rats), and thus it could mainly result from the low density or immature properties of voltage-gated ion channels. We therefore determined whether h-ADF was also found in mature CA3 pyramidal cells. Paired recordings of connected CA3 neurons were obtained in DIV24–DIV32 slice cultures. Brief presynaptic hyperpolarization (200 ms) significantly increased synaptic strength (104.2±1.1% *n*=25; Wilcoxon, *P*<0.01; Supplementary Fig. 2). h-ADF measured in mature cells was smaller from that measured in developing neurons (Mann–Whitney, *P*<0.01; Supplementary Fig. 2). We therefore conclude that h-ADF is developmentally regulated in CA3 neurons *in vitro*.

All recordings were obtained with high extracellular calcium (3 mM) to optimize synaptic strength. Under these conditions, presynaptic release probability is high and presynaptic facilitation such as h-ADF could be underestimated. We therefore measured h-ADF in mature CA3 neurons (DIV24–DIV32) recorded with physiological extracellular calcium (1.3 mM)^22^. Under these conditions, h-ADF was found to be around +16.4% (Wilcoxon, *P*<0.01; Supplementary Fig. 2). We conclude that h-ADF is robustly expressed in mature neurons recorded in physiological extracellular calcium.

### h-ADF is induced by simulated IPSPs and oscillations

**Figure 2:**
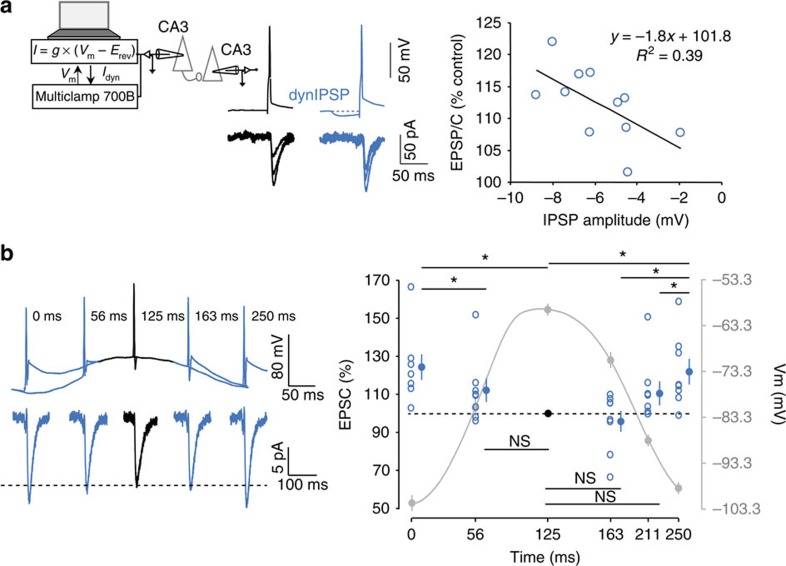
Physiological induction of h-ADF. (**a**) Presynaptic IPSPs induce h-ADF. Left, schematic representation of the system used to inject a dynamic current mimicking a GABAergic input in the presynaptic neuron. Middle, examples of electrophysiological recordings from a connected pair of CA3 neurons in control conditions (black traces) and when a simulated GABAergic input is injected into the presynaptic cell (blue traces). Right, scatter plot showing the normalized EPSP/C as a function of the peak value of the simulated presynaptic IPSP. A clear linear correlation was observed (*y*=−1.8*x*+101.8, Pearson's *R*^2^=0.39, *P*<0.05, *n*=11). (**b**) h-ADF induced during subthreshold *θ* oscillation in CA3 neurons. Left, representative example. Presynaptic spikes are triggered at different phases during a subthreshold oscillation of the membrane potential at 4 Hz. Note that facilitation is observed when the spike is triggered during the hyperpolarized phases of the oscillation. Right, quantitative data (*n*=8). Stars: significant changes (Wilcoxon, *P*<0.05).

To investigate the role of h-ADF under near-physiological conditions, a GABA_A_-like conductance was introduced in the presynaptic neuron using dynamic clamp (Fig. 2a, left). In agreement with results illustrated in Fig. 1, APs preceded by the injection of an IPSC-like current produced a larger response in the postsynaptic neuron compared with APs triggered from resting membrane potential (Wilcoxon *P*<0.001, *n*=11). Consistent with a presynaptic elevation in glutamate release, the PPR was reduced when simulated GABAergic IPSPs preceded APs (from 121% in control to 96%; Wilcoxon *P*<0.05, *n*=7; data not shown). Interestingly, the size of the synaptic potentiation was found to be dependent on the size of the simulated IPSP (*R*^2^=0.39, *P*<0.05), indicating that h-ADF is graded between resting membrane potential (−74 mV) and 10-mV hyperpolarization (−84 mV; Fig. 2a, right). In fact, the facilitation factor in this range was found to be 1.8% per mV of hyperpolarization.

We next investigated the modulation of synaptic strength during presynaptic membrane potential oscillation. Oscillation of the presynaptic membrane potential at 4 Hz was produced by injecting sinusoidal current, and single presynaptic spikes were evoked at different phases of the oscillation. In agreement with the previous results, h-ADF was observed when the cell fired during hyperpolarizing phases of the oscillation (0 ms: 124.3±7%, 250 ms: 122±7%, Wilcoxon *P*<0.05, *n*=8; Fig. 2b). At other phases, the synaptic strength is unchanged (56 ms: 112.2±6%, 163 ms: 95.8±5%, 211 ms: 110.5±6%, Wilcoxon *P*>0.1, *n*=8). In particular, no d-ADF is observed with the depolarization because its duration is too short to inactivate Kv1.1 channels^13^. We conclude that oscillations in the *θ* range induce h-ADF in CA3 neurons.

### h-ADF is associated with an increase in axonal spike amplitude

**Figure 3:**
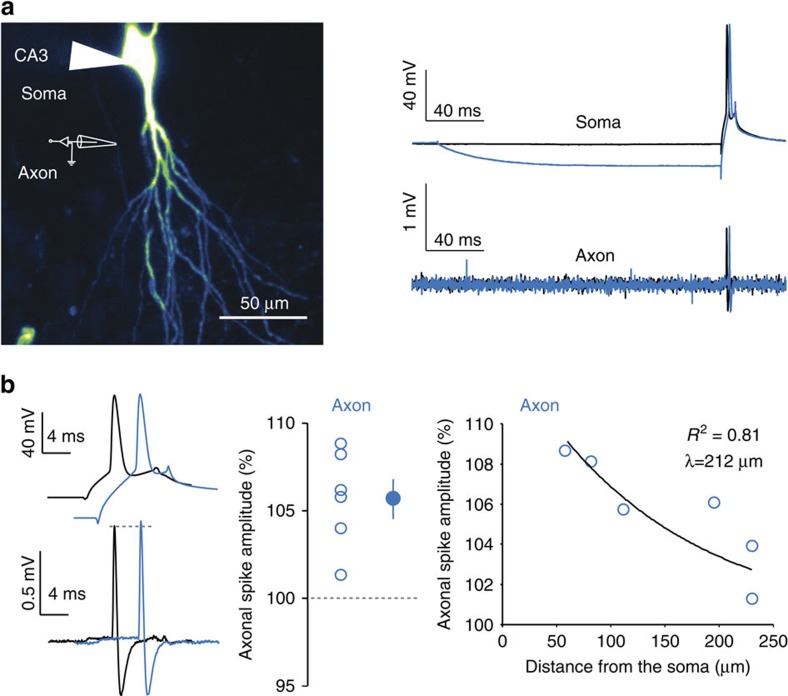
h-ADF enhances spike amplitude in the axon. (**a**) Left, confocal image of a CA3 neuron filled with Alexa 488. The axon collateral (white arrow) is identified on the left and recorded in a cell-attached configuration. Right, simultaneous recordings from the soma (top) and axon (bottom) when the spike is triggered from resting membrane potential (black) or from a transient hyperpolarizing pre-pulse (blue). (**b**) Left, comparison of the spike amplitude measured in the axon evoked with (blue) or without (black) hyperpolarizing pre-pulse. Note the increase in amplitude in the axon when the spike is triggered from the hyperpolarizing pre-pulse. Middle, quantitative analysis of the hyperpolarization-induced enhancement of the axonal spike amplitude in six neurons. Right, scatter plot of the change in the axonal spike amplitude as a function of axonal distance (exponential fit, *y*=11.6e^−*x*/212^, *r*^2^=0.81).

Next, we investigated the mechanisms underlying h-ADF. A possible mechanism for h-ADF is a modulation of the presynaptic spike amplitude induced by the hyperpolarization. We therefore examined the consequence of hyperpolarization on the spike amplitude measured in the axon. CA3 neurons were filled with Alexa 488 (50 μM) to visualize the axon arborization, and cell-attached recordings were obtained from the axon at distances ranging between 60 and 240 μm (Fig. 3a). On somatic hyperpolarization, the amplitude of the axonal spike was enhanced (106±1% of the control amplitude, *n*=6, Wilcoxon, *P*<0.05; Fig. 3b). However, the magnitude of axonal spike facilitation was found to decrease with the axonal distance with a space constant of 212 μm (Fig. 3b). In conclusion, h-ADF in CA3 neurons is associated with a local increase in spike amplitude in the axon.

**Figure 4:**
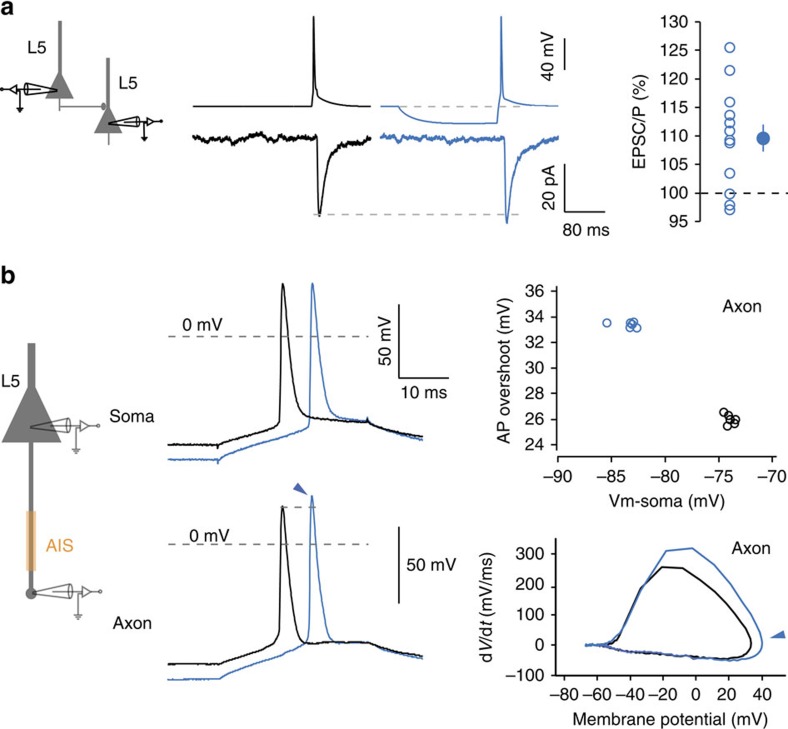
h-ADF at L5–L5 synapses. (**a**) Paired recording of synaptically connected L5 pyramidal neurons. Middle, synaptic facilitation produced by a brief presynaptic hyperpolarization (−20 mV; 200 ms). The EPSCs correspond to averages over 25 traces. Right, h-ADF obtained in 12 L5–L5 pairs. (**b**) Dual soma–axon recordings in L5 pyramidal neurons. Left, experimental design showing double recording from the soma and the axonal bleb of L5 pyramidal neuron. Middle, Soma–axon recording in L5 pyramidal neurons. Note that a brief hyperpolarization of the soma enhances the amplitude of the spike in the axon but not in the soma. Right top, AP overshoot measured in the axon as a function of membrane potential in the cell body, for resting (black) or hyperpolarized (blue) potentials (*n*=6 traces for each case). Right bottom, phase plot of the axonal spikes evoked at rest (black) and following a brief hyperpolarization (blue). Note the enhanced amplitude after a brief hyperpolarization (arrow). The rate of depolarization is also enhanced and the spike threshold is slightly hyperpolarized.

While whole-cell recording from CA3 axons is extremely difficult in organotypic cultures, it can be obtained in L5 pyramidal neurons from acute slices^5^,^6^. Therefore, we first measured whether h-ADF could be also observed at L5–L5 excitatory connections. Pairs of monosynaptically connected L5 pyramidal neurons were recorded in acute slices from the sensori-motor cortex of young rats (P14–P20). Brief hyperpolarization in the soma (200 ms, 10–15 mV) of the presynaptic neuron was found to enhance synaptic strength (109.6±2.3%, *n*=13, Wilcoxon test, *P*<0.05; Fig. 4a).

To confirm that h-ADF in L5 pyramidal neurons was associated with axonal spike amplitude increase, simultaneous whole-cell recordings from the soma and cut-end axons (blebs) were obtained (50–80 μm from the soma) in L5 pyramidal neurons. Transient hyperpolarization of the soma (approximately −13 mV) enhanced the amplitude of the spike overshoot in the axon but not in the soma (+5.5±1.5 versus −0.3±1.1 mV, *n*=5, Mann–Whitney, *P*<0.05; Fig. 4b). The speed of depolarization was also augmented (from 251±59 to 289±56 mV ms^−1^, *n*=5) and the spike threshold was hyperpolarized (from −35.7±5.2 to −38.8±4.3 mV, *n*=5). We conclude that h-ADF in both CA3 and L5 pyramidal cells is associated with the increase in the spike amplitude measured in the axon.

### h-ADF is associated with enhanced axonal calcium signals

**Figure 5:**
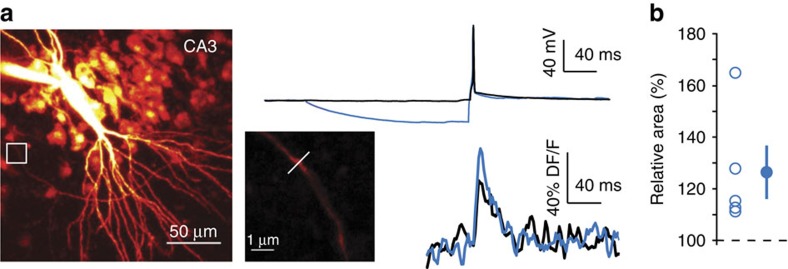
h-ADF enhances spike-evoked calcium signal in the presynaptic terminal of CA3 neurons. (**a**) A brief hyperpolarizing pre-pulse enhances the spike-evoked Ca^2+^ transient. Left top, experimental design showing a CA3 pyramidal neuron filled with Alexa-594 and Fluo-4. White box: area enlarged at right, showing a presynaptic bouton. Right top, voltage traces recorded in the cell body of a CA3 pyramidal neuron. Right bottom, example of fluorescent signals recorded in the presynaptic bouton. The spike-evoked Ca^2+^ transient was increased by ∼20% when the presynaptic spike was evoked following a transient hyperpolarization. (**b**) Quantitative data (*n*=5).

We next used Ca^2+^ imaging to determine the consequence of hyperpolarization-induced enhancement of spike amplitude in the axon. CA3 pyramidal neurons were filled with 50μM Alexa-594; 250 μM Fluo-4 and spike-evoked calcium signals were measured in putative *en passant* boutons at distances ranging between 150 and 250 μm from the soma (Fig. 5a). The integral of the spike-evoked Ca^2+^ transient was increased when the presynaptic spike was evoked following a transient hyperpolarization of ∼20 mV (126±10%, *n*=5; Fig. 5b). We conclude that, during h-ADF, presynaptic hyperpolarization enhances both presynaptic spike amplitude and spike-induced Ca^2+^ influx, which subsequently enhances glutamate release.

### Nav channel inactivation in the axon determines h-ADF

**Figure 6:**
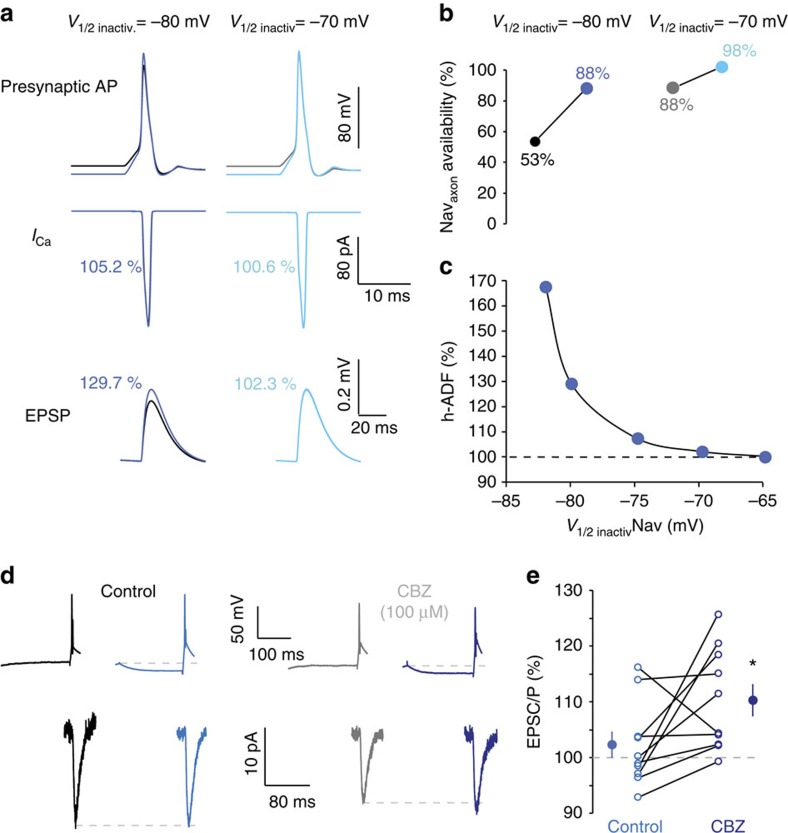
Role of Nav inactivation in h-ADF. (**a**) Simulated h-ADF in control conditions (*V*_1/2_ inactivation=−80 mV for axonal sodium channels). Note the increased amplitude of the spike. Lack of h-ADF when the half-inactivation of the axonal sodium channel is depolarized (*V*_1/2_=−70 mV). (**b**) Summary of the availability of Nav_axon_ with *V*_1/2_ inactivation=−80 mV or −70 mV. Note the marked increase with −80 but not −70 mV. (**c**) Magnitude of simulated h-ADF as a function of *V*_1/2_ inactivation of Nav channels in the axon. Note the increase in h-ADF induced by the hyperpolarization of *V*_1/2_. (**d**) Experimental enhancement of Nav inactivation with CBZ increases the magnitude of h-ADF. Under control condition (left), this connection expresses no h-ADF. When CBZ is added, h-ADF is now visible (right). (**e**) Quantitative data for 10 mature CA3–CA3 connections (DIV 24–32). Star: Wilcoxon, *P*<0.05.

The increased amplitude of the axonal spike during hyperpolarization could be due to the recovery of Nav channels from inactivation. To confirm the role of sodium channel inactivation in h-ADF, we used a NEURON model of two monosynaptically connected CA3 neurons. We then determined the incidence of modifying inactivation of sodium channels in the axon on h-ADF. When the half-inactivation of axonal sodium channels was set to −80 mV (refs 18, 19), somatic hyperpolarization enhanced the spike amplitude, the charge of spike-evoked calcium current and synaptic transmission (Fig. 6a, left). This is due to the recovery of Nav channels from inactivation by hyperpolarization (Fig. 6b, left). However, no change occurred if the half-inactivation of the axonal sodium channels was set to −70 mV (Fig. 6a, right). In this latter case, the proportion of available Nav channels is already very high at resting membrane potential, producing an AP of full amplitude (Fig. 6a,b, right). Therefore, the recovery from inactivation does not further affect the presynaptic spike amplitude. Thus, h-ADF in the model is due to the recovery of Nav channels from inactivation and is increased by hyperpolarizing Nav half-inactivation (Fig. 6c).

To test experimentally this last assumption, we measured h-ADF in the presence of carbamazepine (CBZ, 100 μM), an antiepileptic drug that hyperpolarizes Nav half-inactivation by 6 mV (ref. 23). CBZ reduced spike amplitude (90±2% of control, *n*=10; Supplementary Fig. 3) and synaptic transmission (50±6% of control, *n*=10; Supplementary Fig. 3). As expected, hyperpolarizing Nav inactivation by CBZ increased h-ADF in mature CA3 neurons expressing no h-ADF (from 102.3% in control to 110.3% in the presence of CBZ, *n*=10, Wilcoxon *P*<0.05; Fig. 6d,e).

Moreover, we used our NEURON model to simulate axonal Nav-channel availability during a theta oscillation similar to the one used in Fig. 2b. Nav channels were found to inactivate during depolarization and recover during hyperpolarization, explaining the EPSC modulation during the oscillation (Supplementary Fig. 4). However, inactivation is quicker than recovery during the oscillation because of the slower Nav kinetics at depolarized potentials (Supplementary Fig. 4). This explains why the EPSCs produced at 163 ms did not present any h-ADF, although the spike is emitted from a slightly hyperpolarized potential (Fig. 2b). In fact, at this point of the oscillation Nav channels did not have enough time to recover from inactivation (Supplementary Fig. 4).

Altogether, those results support the fact that h-ADF is due to the recovery of Nav channels from inactivation.

### Nav channel density determines the strength of h-ADF

**Figure 7:**
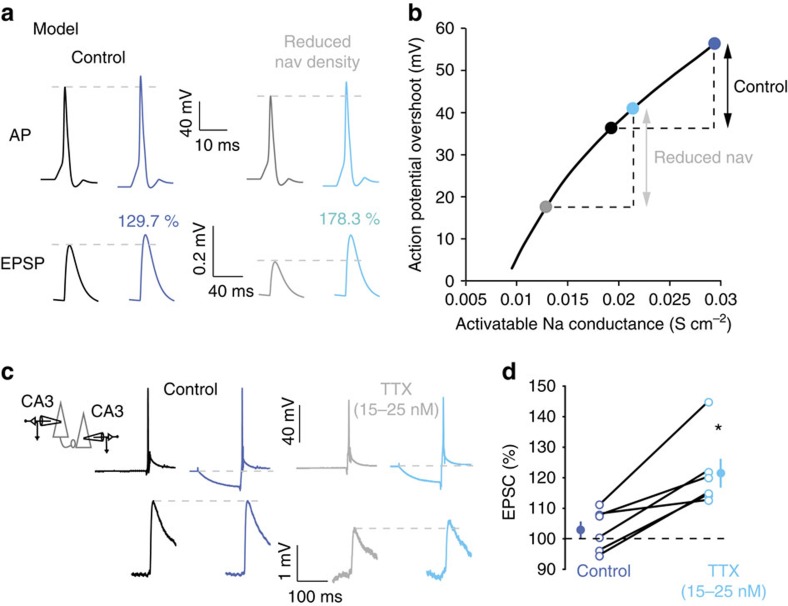
Decreasing Nav channel density with TTX enhances h-ADF. (**a**) Reduction of Nav channel density in the model of h-ADF. Under control conditions (left), h-ADF amounts to +30%. After reducing the Nav channel density (70% of the control, right), h-ADF is increased to +80%. (**b**) Modulation of the presynaptic spike amplitude as a function of activatable Na conductance. Under control conditions, the hyperpolarization from −78 to −93 mV only slightly increases the spike amplitude (black double arrow). When the Nav channel density is reduced, the increase in the spike amplitude is enhanced by 20% (light-blue double arrow). (**c**) Experimental reduction of Nav density with TTX. Under control condition (left), this connection expresses no h-ADF. When a low concentration of TTX is added, transmission is preserved and h-ADF is now visible (right). (**d**) Quantitative data for six mature CA3–CA3 connections (DIV 20–32). Star: Wilcoxon, *P*<0.05.

h-ADF depends on the availability of sodium channels in the axon. Thus, reducing the density of Nav channels should affect h-ADF. In fact, our model showed that reducing Nav channel density to 70% of the control condition enhanced h-ADF from 130 to 180% (Fig. 7a). The critical parameter here was the gain of presynaptic spike overshoot that depends on activatable Na conductance (Fig. 7b). Under control condition, this value was already high, and hyperpolarizing the presynaptic element from −78 to −93 mV enhanced the amplitude of the spike by 28%. When the density of Nav was reduced, the same hyperpolarization enhanced the amplitude of the presynaptic AP by 42%.

We next verified experimentally that reducing Nav channel density increased h-ADF in CA3 neurons. We therefore partially blocked Nav channels with a low concentration of tetrodotoxin (TTX) applied in the bath (15–25 nM). At this concentration TTX blocks ∼80% of the Na^+^ current but preserves induction of fast Na^+^ spikes^24^,^25^. In the presence of TTX, the spike amplitude in the soma was reduced by 45±4% (*n*=9) and synaptic transmission at CA3–CA3 connections was reduced by 55±8% (*n*=9; Supplementary Fig. 5). Most importantly, reducing the proportion of activatable Nav channels with 15–25 nM TTX was found to greatly enhance h-ADF in mature neurons expressing no h-ADF (from 103±3% in control to 121±4% in the presence of TTX, *n*=6, Wilcoxon *P*<0.05; Fig. 7c,d). These data therefore confirm that h-ADF in CA3 neurons depends on the availability of Nav channels.

T-type Ca^2+^ channels are present in the axon. They could be activated during the hyperpolarization–depolarization sequence used to induce h-ADF and thus may account for h-ADF. However, h-ADF was found to remain stable in the presence of 100 nM mibefradil, a T-type channel blocker (from 112.2±1.1% in control to 116.2±11.9% with mibefradil, *n*=3; data not shown), suggesting that T-type Ca^2+^ channels do not participate in h-ADF.

### h-ADF promotes network synchrony

**Figure 8:**
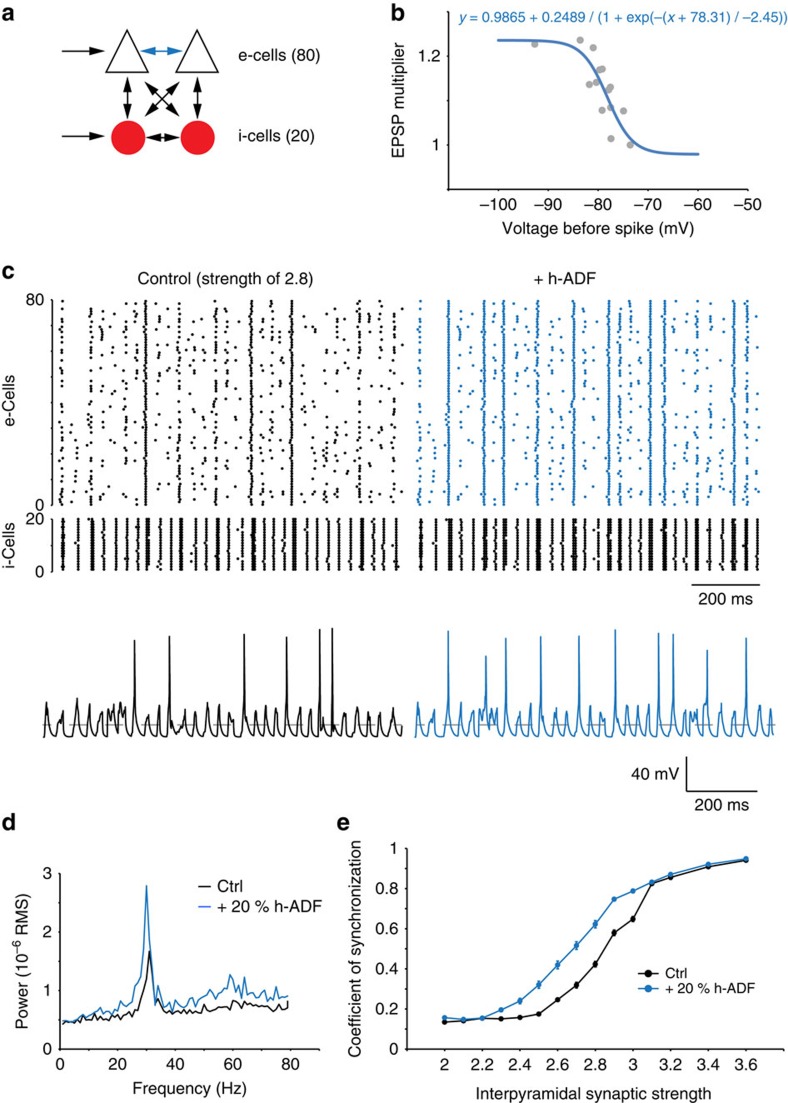
h-ADF promotes network synchrony. (**a**) Scheme of a CA3 network model. The network is composed of 80 e-cells (white triangles) and 20 i-cells (red circles). Pyramidal cells and interneurons were fed by stochastic input. The connections between pyramidal neurons (blue arrows) are the only connections in which h-ADF can be added as h-ADF was not tested experimentally in other connections. (**b**) h-ADF rule at excitatory synapses between pyramidal neurons. A maximal 20% facilitation is applied, according to the membrane voltage measured 17 ms before the spike. (**c**) Effect of the h-ADF rule on network synchrony. Left top, rastergram showing the network activity in control conditions with a synaptic strength of 2.8 mS. Left bottom, representative trace in an e-cell. Right top, with the h-ADF rule (+20% h-ADF), the synchrony is increased. Right bottom, representative trace in an e-cell. Note that membrane potential crosses the −73-mV limit between spikes (dotted lines). (**d**) Power spectrum of the data shown in **c** (synaptic strength of 2.8 mS). Adding h-ADF rules dramatically increases the network synchrony around the gamma frequency (29 Hz). (**e**) Synchronization coefficients calculated for synaptic strengths from 2 to 3.6. Incorporation of h-ADF increases synchrony (blue).

We next tested the implication of h-ADF in network synchrony using a hippocampal network model formed by 80 pyramidal-like excitatory cells (e-cells) and 20 interneuron-like inhibitory cells (i-cells) interconnected (Fig. 8a; see Methods). e- and i-cells were fed by stochastic input. The network of e-cells became synchronized, and oscillations in the gamma range appeared as synaptic strength between e-cells increased (Supplementary Fig. 6). These oscillations were driven by i-cells: activation of e-cells was found to promote the activation of i-cells, which in turn silenced the whole network (Supplementary Fig. 6). Since h-ADF increases interpyramidal synaptic strength when the presynaptic spike is preceded by an IPSP, h-ADF is a good candidate to promote these i-cell-driven oscillations.

The h-ADF rule was incorporated in the network by increasing synaptic strength between e-cells according to the membrane potential measured 17 ms before the spike. In fact, synaptic strength was increased by 20% if the presynaptic potential was below −84 mV (Fig. 8b). This rule was directly derived from values measured experimentally (see Figs 1a and 2a). For an e-cell-synaptic strength of 2.8 mS, adding h-ADF in the network markedly enhanced both the firing frequency and synchrony across e-cells (Fig. 8c–e). In fact, the propensity to oscillate in the gamma range was greatly facilitated if h-ADF between e-cells was effective (Fig. 8e). Interestingly, in a network with shunting inhibition (*E*_Cl_=−73 mV instead of −80 mV in control condition), h-ADF rule did not improve synchrony and did not promote gamma oscillations (Supplementary Fig. 6). However, as h-ADF increases the synaptic strength between e-cells, its synchronizing effect could be simply due to the increase in the spike rate of the network. To increase the spike rate without affecting synaptic strength, we decided to fix the inter-e-cell strength at 2.5 mS and increase the external drive frequency of e-cells from 6 to 20 Hz. We plotted the coefficient of synchronization versus the spike rate of the network. Even if synchrony showed to be linearly correlated to spike rate, h-ADF increased the coefficient of synchronization for any given spike rate in the 4–14-Hz range (Supplementary Fig. 6). This showed that for low spike rate, h-ADF increases synchrony independently of the mean network activity. In conclusion, in our model, h-ADF increases network synchronization and promotes oscillations by linking interpyramidal synaptic strength with activity of interneurons.

## Discussion

We report here a physiologically relevant mode of AD regulation of synaptic transmission at CA3–CA3 and L5–L5 connections. Synaptic transmission was found to be enhanced by ∼20% owing to brief physiological hyperpolarization (15–200 ms) preceding the presynaptic AP. h-ADF is associated with a gain in AP amplitude measured in the axon, and is modulated by pharmacological agents targeting Nav channels. Thus, h-ADF can be considered as a novel form of activity-dependent synaptic plasticity that depends on Nav channels. Although h-ADF was found to be developmentally downregulated, mature CA3 neurons still expressed robust h-ADF (16%) in physiological calcium (1.3 mM). h-ADF is mediated by an increase in presynaptic glutamate release. It can be induced by IPSP-like synaptic conductance or theta-like oscillations. Integrated in a network model, h-ADF promotes network synchrony and favours the emergence of gamma oscillations.

h-ADF is an unanticipated mode of AD facilitation of excitatory synaptic transmission, induced by a brief hyperpolarization of the presynaptic cell. While analogue modulation of spike-evoked synaptic transmission had been reported to occur in many neuronal types following presynaptic depolarization^1^,^5^,^6^,^8^,^10^,^12^,^13^, it was not entirely clear whether presynaptic hyperpolarization robustly induced synaptic facilitation^26^,^27^,^28^. In these latter studies the underlying mechanisms were not identified. We characterize here for the first time the induction of h-ADF in CA3 and L5 pyramidal neurons. This facilitation was found to fully develop with moderate hyperpolarization. In fact, a 10-mV hyperpolarization from resting membrane potential was found to be sufficient to enhance synaptic transmission by ∼20% at CA3–CA3 synapses, indicating that the facilitation factor was ∼2% per mV of hyperpolarization. Interestingly, facilitation was not further enhanced by a larger hyperpolarization, suggesting that the presynaptic Na^+^ spike reaches its maximal amplitude with a small fraction of available Nav channels^24^. In addition, simulated IPSPs preceding the spike by 50 ms enhanced synaptic transmission in a graded manner.

We show that h-ADF and d-ADF are coexpressed at CA3–CA3 pyramidal cell synapses. A combination of stimuli that separately induced d-ADF and h-ADF produced facilitation that was significantly greater, indicating that the two processes are mechanistically distinct. In fact, d-ADF depends on Kv1.1 and h-ADF depends on Nav channels. In contrast to d-ADF that requires several seconds to be fully expressed^5^,^6^,^13^, h-ADF can be induced by fast (∼15 ms) hyperpolarization of the presynaptic neuron. Thus, h-ADF is better suited than d-ADF for fast dynamics of network activity.

h-ADF is observed at both hippocampal CA3 cells and L5 pyramidal neurons, indicating that it might be a general process in local synaptic circuits. h-ADF seems to be developmentally regulated in hippocampal CA3 cells and declines from young CA3 neurons to mature neurons. The developmental modulation may result from the increase in the density of voltage-gated sodium channels in the axon^29^,^30^,^31^,^32^. However, under physiological conditions (1.3 mM external Ca^2+^), h-ADF measured at mature CA3–CA3 synapses is robust and amounts to 16%.

Two mechanisms of analogue modulation of spike-evoked synaptic transmission have been identified so far. Release can be elevated by voltage inactivation of Kv1 channels, making the presynaptic spike broader^5^,^6^,^13^ or by voltage activation of P/Q-type Ca^2+^ channels that elevate basal calcium^8^,^9^. While the underlying mechanisms are different, these forms of ADF share common characteristics: they are depolarization-driven and need a long depolarization (hundreds of milliseconds to few seconds) to be fully expressed. Here we show that ADF can also be induced by presynaptic *hyperpolarization*. This hyperpolarization propagates passively along the axon to allow the recovery of Nav channel from inactivation. In turn, it increases AP amplitude, thus enhancing Ca^2+^ entry in the presynaptic bouton and subsequently elevating glutamate release. All these steps have been dissected here with the use of electrophysiological, pharmacological, imaging and computational approaches.

The major consequence of presynaptic hyperpolarization is the enhancement of spike amplitude in the axon. This enhancement was reported in both CA3 and L5 neurons using cell-attached or whole-cell recordings, respectively. It is supposed to result from the fast recovery of Nav channels from inactivation. Although Na^+^ channel inactivation has not been directly measured in our study, we assume that, at resting membrane potential, a very large proportion of Na^+^ channels are inactivated in the axon of CA3 pyramidal cells, as in neocortical pyramidal neurons and hippocampal granule cells^18^,^19^,^33^. As predicted by cable theory, the modulation in extracellular spike amplitude was found to decline with the axonal distance. The space constant here has been estimated to be ∼212 μm. Compared with d-ADF, this space constant is slightly shorter probably because of the frequency dependence of the space constant along axons^5^,^34^. However, h-ADF could affect more distal synapses in CA1 or L5 pyramidal neurons that express long axonal space constant (700–1,000 μm)^34^,^35^.

The involvement of Nav channels was shown by modulating their availability. The model predicted that decreasing the density of Nav channels would increase the hyperpolarization-induced modulation of the presynaptic AP amplitude and it would subsequently increase h-ADF. Experimentally, low concentrations of TTX (15–25 nM) increased h-ADF by a factor 5 (Fig. 7). In addition, our model showed that hyperpolarizing the half-inactivation of Nav channel in the axon enhanced the hyperpolarization-induced modulation of the spike amplitude and subsequently increased h-ADF. Further supporting the contribution of Nav channel in h-ADF, we show that hyperpolarizing the inactivation properties of sodium channels with CBZ also increased h-ADF.

The Nav channel-dependent enhancement of spike amplitude induced by hyperpolarization caused an elevation in presynaptic calcium. In fact, a brief hyperpolarization before the AP increased the evoked calcium signal measured in putative presynaptic boutons. A hyperpolarization indeed increased AP amplitude, leading to a larger calcium influx in the bouton and thus a bigger postsynaptic response. These results were reproduced in our model and are consistent with previous studies^36^,^37^.

Presynaptic transient hyperpolarization has a major consequence on fast activating or inactivating currents. Except the recovery of Nav channels from inactivation, the biophysical state of Cav channels might, in principle, be also altered. h-ADF is not suppressed by mibefradil, a selective blocker of T-type Ca^2+^ channels. In addition, h-ADF does not result from the increase in driving force for Ca^2+^ because the hyperpolarization occurs before and not during the presynaptic AP.

A major functional consequence of h-ADF reported in our study is the optimization of synaptic excitation in local circuits by IPSPs preceding presynaptic APs. Thus, our results strongly suggest that synaptic transmission between pyramidal cells that fire in rebound on activation of basket cells could be facilitated^38^, subsequently enhancing the coherence of network oscillations. The importance of h-ADF in brain function is supported by several lines of evidence. First, biologically realistic hyperpolarization such as GABA_A_-like IPSPs simulated using the dynamic-clamp induced robust h-ADF. In this case, the hyperpolarization was small (2–10 mV) and the delay between the IPSP and the spike was in the physiological range (50 ms). Similarly, presynaptic oscillation of the membrane potential was found to induce h-ADF when the spike was triggered during the hyperpolarized part of the oscillation. In this case, h-ADF can be seen as a normalizing effect: during the depolarizing phase of the oscillation, the probability to trigger a spike is high and the postsynaptic response is low, whereas during the hyperpolarizing phase of the oscillation the spike probability is low and the response is high. We performed our experiments for a 4-Hz oscillation; however, given the quick kinetics of h-ADF (a 15-ms hyperpolarization is enough to fully induce it), it should be observed for higher theta rhythms (8–12 Hz).

A particular important result of our study is the effect of h-ADF on a model of network activity. In fact, h-ADF improved network synchrony and promoted gamma oscillation in a simple interneuron-like-driven network model. Incorporated at synapses established between pyramidal-like cells, h-ADF increased the global synchrony of the network and promoted the emergence of gamma oscillation. It is important to notice that h-ADF also acts as a mechanism optimizing biological energy. A given synchrony is obtained with less excitatory synaptic strength. The specificity of the effect was demonstrated by making inhibitory synapses shunting instead of hyperpolarizing. In this case, the presence of h-ADF had no effect. These results strongly suggest that Nav channel modulation of synaptic efficacy is probably a major mechanism for modulating information processing in local neural circuits.

## Methods

### Organotypic cultures of the rat hippocampus

Organotypic cultures of the rat hippocampus were prepared^21^, according to the European and Institutional guidelines (Council Directive 86/609/EEC and French National Research Council and approved by the local health authority (# D 13 055 08, Préfecture des Bouches-du-Rhône, Marseille)). Postnatal day 5–7 Wistar rats of both sexes were anaesthetized with chloral hydrate injection, the brain was rapidly extracted from the skull and each hippocampus was isolated. Hippocampal slices (350 μm) were maintained for up to 30–35 days in an incubator at 34 °C, 95% O_2_–5% CO_2_. The culture medium contained (in ml) 25 MEM, 12.5 HBSS, 12.5 horse serum, 0.5 penicillin (10,000 U ml^−1^), 0.5 streptomycin (10,000 μg ml^−1^), 0.8 glucose (1 M), 0.1 ascorbic acid (1 mg ml^−1^), 0.4 HEPES (1 M), 0.5 B27 and 8.95 sterile H_2_O. To avoid glial proliferation, 5 μM Ara-C was added to the medium starting at 3 DIV. Pyramidal cells from CA3 area were recorded at the age of 8–12 DIV or 24–32 DIV when mentioned.

### Acute slices of the rat neocortex

Neocortical slices (350–400 μm) were obtained from 14- to 20-day-old Wistar rats of both sexes. Rats were deeply anaesthetized with chloral hydrate (intraperitoneal, 200 mg kg^−1^) and killed by decapitation. Slices were cut in an ice-cold solution containing (mM): 280 sucrose, 26 NaHCO_3_, 10 D-glucose, 10 MgCl_2_, 1.3 KCl and 1 CaCl_2_, and were bubbled with 95% O_2_–5% CO_2_, pH 7.4. Slices were recovered (1 h) in a solution containing (mM): 125 NaCl, 26 NaHCO_3_, 2 CaCl_2_, 2.5 KCl, 2 MgCl_2_, 0.8 NaH_2_PO_4_ and 10 D-glucose, and were equilibrated with 95% O_2_–5% CO_2_. Each slice was transferred to a submerged chamber mounted on an upright microscope (Olympus BX51WI or Zeiss LSM-710), and neurons were visualized using differential interference contrast infrared videomicroscopy.

### Paired recordings and analysis

Dual recordings from pairs of neurons were obtained as previously described^21^,^39^. The external saline contained (in mM): 125 NaCl, 26 NaHCO_3_, 3 CaCl_2_ (1.3 for physiological calcium solution), 2.5 KCl, 2 MgCl_2_ (1 for physiological calcium solution), 0.8 NaH_2_PO_4_ and 10 D-glucose, and was equilibrated with 95% O_2_–5% CO_2_. Patch pipettes (5–10 MΩ) were pulled from borosilicate glass and filled with an intracellular solution containing (mM): 120 K gluconate, 20 KCl, 10 HEPES, 0.5 EGTA, 2 MgCl_2_, 2 Na_2_ATP and 0.3 NaGTP (pH 7.4). Recordings were performed at 30 °C in a temperature-controlled recording chamber (Luigs & Neumann, Ratingen, Germany). Usually, the presynaptic neuron was recorded in current clamp and the postsynaptic cell in voltage clamp. Both pre- and postsynaptic cells were held at their resting membrane potential (approximately −77 mV). Presynaptic APs were generated by injecting brief (5 ms) depolarizing pulses of current at a frequency of 0.1 Hz. PPR was assessed with two presynaptic stimulations (50-ms interval). Voltage and current signals were low-pass-filtered (3 kHz), and sequences (200–500 ms) were acquired at 10–20 kHz with pClamp (Axon Instruments, Molecular Devices) version 10. Electrophysiological signals were analysed with ClampFit (Axon Instruments) and custom-made softwares written in LabView (National Instruments). Postsynaptic responses could be averaged following alignment of the presynaptic APs using automatic peak detection. The presence or absence of a synaptic connection was determined on the basis of averages of 30–50 individual traces. TTX (Latoxan, 15–25 nM) was bath-applied. The membrane potential was corrected for the liquid junction potential (−13 mV). Pooled data are represented as mean±s.e. in all the figures, and we used the Mann–Whitney *U*-test or Wilcoxon rank-signed test for statistical comparison.

### Calcium imaging

CA3 pyramidal neurons were imaged using a LSM710 Zeiss confocal system. For imaging calcium in CA3 neurons, 50 μM Alexa-594 and 250 μM Fluo-4 (Invitrogen) were added to the pipette solution. Alexa-594 fluorescence was used to reveal neuronal morphology, whereas fluorescence signals emitted by Fluo-4 were used for calcium imaging.

Laser sources for fluorescence excitation were set at 488 nm for Fluo-4 and 543 nm for Alexa-594. Emitted fluorescence was collected between 500 and 580 nm for Fluo-4, and between 620 and 750 nm for Alexa-594. After whole-cell access, the dyes diffused at least 15–20 min before measuring fluorescence. APs were induced by short (5–10 ms) pulses of depolarizing current pulses (900–1,400 pA) in the current clamp mode and were synchronized with calcium imaging in the line-scan mode. Depending on experiments, line-scan speed was set to 0.5–2 kHz.

Acquired Fluo-4 fluorescence signals were converted to Δ*F*/*F* values. Peak amplitude and decay time of Ca^2+^ signals were calculated with a custom-made analysis programme written in LabView (National Instruments).

The axon was identified visually as a long, thin, aspiny process with constant diameter, emerging from the soma or from a primary dendrite near the cell body. The axon was followed until the first bulges of the membrane, revealing presynaptic boutons, were encountered.

### Axonal recordings

Two modes of axonal recordings were used in this study. First, simultaneous recordings from the soma in whole-cell configuration and the axon in cell-attached configuration were obtained from CA3 pyramidal neurons as previously described^25^. The axon was recorded for 10–15 min after whole-cell access of the somatic compartment and the full labelling of the axon by Alexa 488 and visualized with the LSM-710 confocal microscope. To obtain cell-attached recording, a brief suction was applied to the pipette. Under these conditions, the spike measured in the axon had a positive polarity (amplitude 0.3–1.2 mV).

The second type of axonal recording was obtained in the whole-cell configuration. Simultaneous recordings from the soma and axonal cut ends (blebs, at 50–80 μm from the cell body) were obtained in L5 pyramidal neurons. In that case, identification of the axonal bleb was made visually at the surface of the acute slice.

### Dynamic clamp

Synaptic-ADF experiments (Fig. 2a) were performed using a dynamic-clamp system (SM-1; Cambridge Conductance, Cambridge, UK) and a computer-generated conductance profile delivered via AD interface (Digidata 1440A; Molecular Devices). The conductance profile was based on recordings of GABAergic currents obtained from CA3 neurons in voltage clamp while stimulating extracellularly the pyramidal layer in the presence of 2 mM kynurenate (Sigma). The conductance waveform follows a double exponential profile:

*y*=−(1−exp(−(*t*/*t*_r_))) × exp(−(*t*/*t*_d_)), where *t*_r_=4.2 ms and *t*_d_=14.5 ms (determined experimentally). The reversal potential for the GABAergic-like current was set at −86 mV for this specific experiment.

### Modelling

A multicompartment model of a CA3 pyramidal neuron was simulated with NEURON 7.2. The diameter and length of each compartment are provided in Supplementary Table 1. The passive electrical properties *C*_m_ and *R*_i_ were set to 1 μF cm^−2^ and 100 Ω cm, respectively, uniformly throughout all compartments^40^. All simulations were run with 10-μs time steps and the nominal temperature of simulations was 28 °C. The voltage dependence of activation and inactivation of a Hodgkin–Huxley-based conductance models (*g*_NaSoma_, *g*_NaAxon_, *g*_KDR_, *g*_Kv1_ and *g*_Ca_) are given as follows:


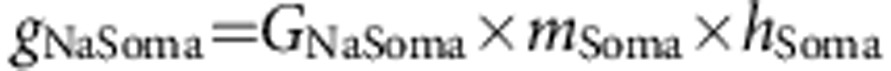



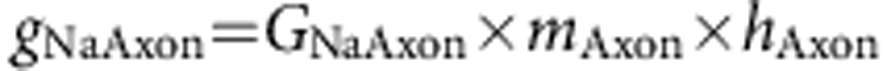



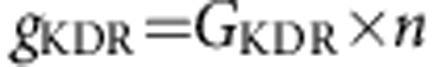



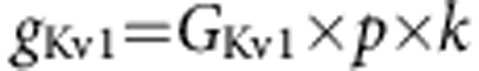



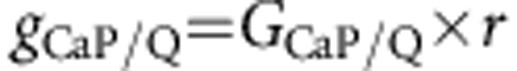


where *m*_Soma_, *m*_Axon_, *n*, *p* and *r* are dynamic activation variables and *h*_Soma_, *h*_Axon_ and *k* are dynamic inactivation variables. They evolve according to the following differential equations (adapted from ref. 19 for Nav_Soma_ and Nav_Axon_; ref. 41 for K_DR_; ref. 42 for *K*_v_1 and ref. 36 for CaP/Q):


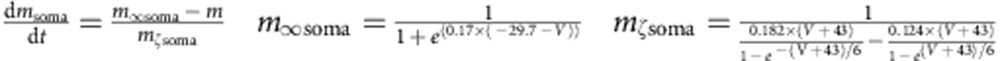



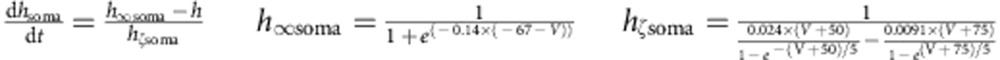



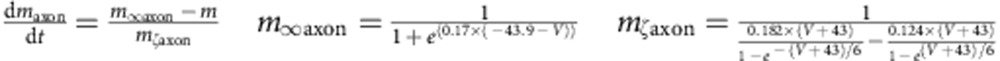



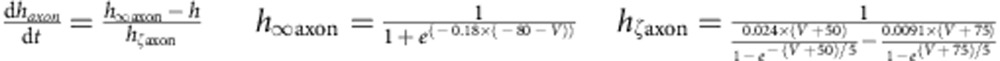



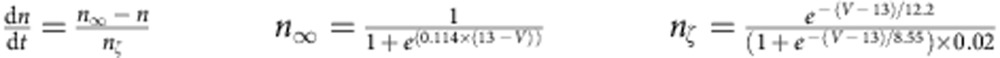



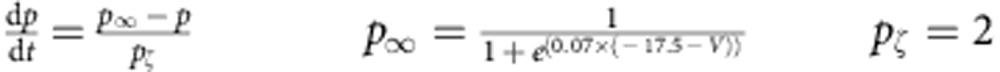



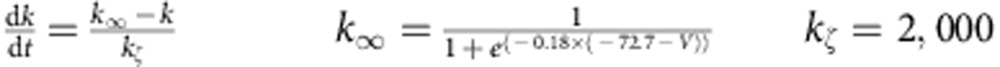



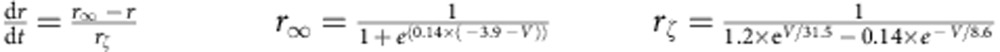


where *V* is the membrane potential of the simulated neuron. The equilibrium potentials for Na^+^, K^+^, Ca^2+^ and passive channels were set to +93, −105, +130 and −50 mV, respectively. The conductance density is provided in Supplementary Table 1.

The resting membrane potential was set to −78 mV in control condition and to −93 mV for the hyperpolarized state. These variations in somatic resting potential propagated along the axon with a space constant of 200 μm.

### Model of neuronal network

We developed a model of neuronal network inspired by the model described in ref. 43 (https://senselab.med.yale.edu; Model DB accession no. 138421). Here the conductance values in pyramidal neurons were replaced by our Na_Soma_ and *K*_DR_ conductance values (see above). The reversal potential for GABA_A_ was set to −80 mV (ref. 44). This value is a hyperpolarized estimate of EGABA_A_ but is still in the physiological range.

The connections between pyramidal neurons (e-cells, e) and interneurons (i-cells, i) are described by two parameters, respectively, the probability of connection between the different cells, *p*, and the strength of connection, *g_hat*. In our model, these parameters were set as *p*_ee=1, p_ei=1, p_ie=0.5, p_ii=1; *g_hat*_ee varying from 2 to 3.6 mS, *g_hat*_ei=1 mS, g_hat_ie=1.8 mS and *g_hat*_ii=0.5 mS.

The network was driven by one stochastic input on e-cells and one on i-cells. Stochastic inputs are characterized by their frequency *f_stoch*, their strength *g_stoch* and their decay time *tau_d_stoch*. We used *f_stoch*_e=10 Hz, *g_stoch*_e=0.5 mS, *tau_d_stoc*h_e=3 ms and *f_stoch*_i=5 Hz, *g_stoch*_i=0.1 mS, *tau_d_stoch*_i=3 ms for stochastic inputs on e- and i-cells, respectively.

Simulations ran for 1-s duration, with a time interval of 0.02 ms. To set the h-ADF rule, we saved the voltage of each cell in an independent matrix and multiplied it by the equation: *Y*=0.9865+0.2489/(1+exp(−(V17+78.31)/−2.45)). *Y* being the h-ADF multiplier depending on V17, the voltage of the cell was 17 ms before the spike. The excitatory synaptic input between e-cells was then multiplied by this factor.

We ran 20 simulations for every value of *g_hat*_ee from 2 to 3.6 mS, and then calculated the coefficient of synchronization for every rastergram obtained. The coefficient of synchronization *k*(*τ*) was calculated as described in ref. 45 as an average value of coefficients *k*_i,j_ (*τ*) for each pair of e-cells (i, j) in the network. The time window was divided into bins of *τ*=1/*f*_network_, with *f*_network_ as the average frequency of the network, and APs were represented in a binary format (spike/no-spike pattern).

The network simulation was run on Matlab 2011 (MathWorks), and analysis of the coefficient of synchronization was performed with the custom software written in Labview 2010 (National Instruments).

## Additional information

**How to cite this article:** Rama, S. *et al.* Presynaptic hyperpolarization induces a fast analogue modulation of spike-evoked transmission mediated by axonal sodium channels. *Nat. Commun.* 6:10163 doi: 10.1038/ncomms10163 (2015).

## Supplementary Material

Supplementary Information Supplementary Figures 1-6 and Supplementary Table 1

## References

[b1] Shimahara T. & Tauc L. Multiple interneuronal afferents to the giant cells in Aplysia. J. Physiol. 247, 299–319 (1975) . 1151777 10.1113/jphysiol.1975.sp010933 PMC1309471

[b2] Shimahara T. & Peretz B. Soma potential of an interneurone controls transmitter release in a monosynaptic pathway in Aplysia. Nature 273, 158–160 (1978) . 25388 10.1038/273158a0

[b3] Nicholls J. & Wallace B. G. Modulation of transmission at an inhibitory synapse in the central nervous system of the leech. J. Physiol. 281, 157–170 (1978) . 212550 10.1113/jphysiol.1978.sp012414 PMC1282689

[b4] Shapiro E., Castellucci V. F. & Kandel E. R. Presynaptic membrane potential affects transmitter release in an identified neuron in Aplysia by modulating the Ca2^+^ and K^+^ currents. Proc. Natl Acad. Sci. USA 77, 629–633 (1980) . 6244571 10.1073/pnas.77.1.629 PMC348328

[b5] Shu Y., Hasenstaub A., Duque A., Yu Y. & McCormick D. A. Modulation of intracortical synaptic potentials by presynaptic somatic membrane potential. Nature 441, 761–765 (2006) . 16625207 10.1038/nature04720

[b6] Kole M. H., Letzkus J. J. & Stuart G. J. Axon initial segment Kv1 channels control axonal action potential waveform and synaptic efficacy. Neuron 55, 633–647 (2007) . 17698015 10.1016/j.neuron.2007.07.031

[b7] Zhu J., Jiang M., Yang M., Hou H. & Shu Y. Membrane potential-dependent modulation of recurrent inhibition in rat neocortex. PLoS Biol. 9, e1001032 (2011) . 21445327 10.1371/journal.pbio.1001032 PMC3062529

[b8] Christie J. M., Chiu D. N. & Jahr C. E. Ca(2^+^)-dependent enhancement of release by subthreshold somatic depolarization. Nat. Neurosci. 14, 62–68 (2011) . 21170054 10.1038/nn.2718 PMC3130502

[b9] Bouhours B., Trigo F. F. & Marty A. Somatic depolarization enhances GABA release in cerebellar interneurons via a calcium/protein kinase C pathway. J. Neurosci. 31, 5804–5815 (2011) . 21490222 10.1523/JNEUROSCI.5127-10.2011 PMC6622834

[b10] Alle H. & Geiger J. R. Combined analog and action potential coding in hippocampal mossy fibers. Science 311, 1290–1293 (2006) . 16513983 10.1126/science.1119055

[b11] Scott R., Ruiz A., Henneberger C., Kullmann D. M. & Rusakov D. A. Analog modulation of mossy fiber transmission is uncoupled from changes in presynaptic Ca2^+^. J. Neurosci. 28, 7765–7773 (2008) . 18667608 10.1523/JNEUROSCI.1296-08.2008 PMC2685171

[b12] Sasaki T., Matsuki N. & Ikegaya Y. Effects of axonal topology on the somatic modulation of synaptic outputs. J. Neurosci. 32, 2868–2876 (2012) . 22357869 10.1523/JNEUROSCI.5365-11.2012 PMC6621900

[b13] Bialowas A. *et al.* Analog modulation of spike-evoked transmission in CA3 circuits is determined by axonal Kv1.1 channels in a time-dependent manner. Eur. J. Neurosci. 41, 293–304 (2015) . 25394682 10.1111/ejn.12787

[b14] Awatramani G. B., Price G. D. & Trussell L. O. Modulation of transmitter release by presynaptic resting potential and background calcium levels. Neuron 48, 109–121 (2005) . 16202712 10.1016/j.neuron.2005.08.038

[b15] Debanne D., Bialowas A. & Rama S. What are the mechanisms for analogue and digital signalling in the brain? Nat. Rev. Neurosci. 14, 63–69 (2013) . 23187813 10.1038/nrn3361

[b16] Shu Y., Yu Y., Yang J. & McCormick D. A. Selective control of cortical axonal spikes by a slowly inactivating K^+^ current. Proc. Natl Acad. Sci. USA 104, 11453–11458 (2007) . 17581873 10.1073/pnas.0702041104 PMC2040919

[b17] Shu Y., Duque A., Yu Y., Haider B. & McCormick D. A. Properties of action-potential initiation in neocortical pyramidal cells: evidence from whole cell axon recordings. J. Neurophysiol. 97, 746–760 (2007) . 17093120 10.1152/jn.00922.2006

[b18] Engel D. & Jonas P. Presynaptic action potential amplification by voltage-gated Na^+^ channels in hippocampal mossy fiber boutons. Neuron 45, 405–417 (2005) . 15694327 10.1016/j.neuron.2004.12.048

[b19] Hu W. *et al.* Distinct contributions of Na(v)1.6 and Na(v)1.2 in action potential initiation and backpropagation. Nat. Neurosci. 12, 996–1002 (2009) . 19633666 10.1038/nn.2359

[b20] Schmidt-Hieber C. & Bischofberger J. Fast sodium channel gating supports localized and efficient axonal action potential initiation. J. Neurosci. 30, 10233–10242 (2010) . 20668206 10.1523/JNEUROSCI.6335-09.2010 PMC6633381

[b21] Debanne D. *et al.* Paired-recordings from synaptically coupled cortical and hippocampal neurons in acute and cultured brain slices. Nat. Protoc. 3, 1559–1568 (2008) . 18802437 10.1038/nprot.2008.147

[b22] Jones H. C. & Keep R. F. Brain fluid calcium concentration and response to acute hypercalcaemia during development in the rat. J. Physiol. 402, 579–593 (1988) . 3236250 10.1113/jphysiol.1988.sp017223 PMC1191910

[b23] Niespodziany I. *et al.* Brivaracetam differentially affects voltage-gated sodium currents without impairing sustained repetitive firing in neurons. CNS Neurosci. Ther. 21, 241–251 (2015) . 25444522 10.1111/cns.12347 PMC4359682

[b24] Madeja M. Do neurons have a reserve of sodium channels for the generation of action potentials? A study on acutely isolated CA1 neurons from the guinea-pig hippocampus. Eur J. Neurosci. 12, 1–7 (2000) . 10651854 10.1046/j.1460-9568.2000.00871.x

[b25] Boudkkazi S., Fronzaroli-Molinieres L. & Debanne D. Presynaptic action potential waveform determines cortical synaptic latency. J. Physiol. 589, 1117–1131 (2011) . 21224227 10.1113/jphysiol.2010.199653 PMC3060591

[b26] Takeuchi A. & Takeuchi N. Electrical changes in pre- and postsynaptic axons of the giant synapse of Loligo. J. Gen. Physiol. 45, 1181–1193 (1962) . 13919241 10.1085/jgp.45.6.1181 PMC2195243

[b27] Thio L. L. & Yamada K. A. Differential presynaptic modulation of excitatory and inhibitory autaptic currents in cultured hippocampal neurons. Brain Res. 1012, 22–28 (2004) . 15158157 10.1016/j.brainres.2004.02.077

[b28] Cowan A. I. & Stricker C. Functional connectivity in layer IV local excitatory circuits of rat somatosensory cortex. J. Neurophysiol. 92, 2137–2150 (2004) . 15201316 10.1152/jn.01262.2003

[b29] Gong B., Rhodes K. J., Bekele-Arcuri Z. & Trimmer J. S. Type I and type II Na(^+^) channel alpha-subunit polypeptides exhibit distinct spatial and temporal patterning, and association with auxiliary subunits in rat brain. J. Comp. Neurol. 412, 342–352 (1999) . 10441760

[b30] Liao Y. *et al.* Molecular correlates of age-dependent seizures in an inherited neonatal-infantile epilepsy. Brain 133, 1403–1414 (2010) . 20371507 10.1093/brain/awq057

[b31] Nigro M. J., Quattrocolo G. & Magistretti J. Distinct developmental patterns in the expression of transient, persistent, and resurgent Na^+^ currents in entorhinal cortex layer-II neurons. Brain Res. 1463, 30–41 (2012) . 22608073 10.1016/j.brainres.2012.04.049

[b32] Gutzmann A. *et al.* A period of structural plasticity at the axon initial segment in developing visual cortex. Front Neuroanat 8, 11 (2014) . 24653680 10.3389/fnana.2014.00011 PMC3949221

[b33] Colbert C. M. & Pan E. Ion channel properties underlying axonal action potential initiation in pyramidal neurons. Nat. Neurosci. 5, 533–538 (2002) . 11992119 10.1038/nn0602-857

[b34] Christie J. M. & Jahr C. E. Selective expression of ligand-gated ion channels in L5 pyramidal cell axons. J. Neurosci. 29, 11441–11450 (2009) . 19759293 10.1523/JNEUROSCI.2387-09.2009 PMC2814532

[b35] Kim S. Action potential modulation in CA1 pyramidal neuron axons facilitates OLM interneuron activation in recurrent inhibitory microcircuits of rat hippocampus. PLoS ONE 9, e113124 (2014) . 25409299 10.1371/journal.pone.0113124 PMC4237399

[b36] Bischofberger J., Geiger J. R. & Jonas P. Timing and efficacy of Ca2^+^ channel activation in hippocampal mossy fiber boutons. J. Neurosci. 22, 10593–10602 (2002) . 12486151 10.1523/JNEUROSCI.22-24-10593.2002 PMC6758411

[b37] Berger C., Meyer E. M., Ammer J. J. & Felmy F. Large somatic synapses on neurons in the ventral lateral lemniscus work in pairs. J. Neurosci. 34, 3237–3246 (2014) . 24573282 10.1523/JNEUROSCI.3664-13.2014 PMC6795299

[b38] Cobb S. R., Buhl E. H., Halasy K., Paulsen O. & Somogyi P. Synchronization of neuronal activity in hippocampus by individual GABAergic interneurons. Nature 378, 75–78 (1995) . 7477292 10.1038/378075a0

[b39] Boudkkazi S. *et al.* Release-dependent variations in synaptic latency: a putative code for short- and long-term synaptic dynamics. Neuron 56, 1048–1060 (2007) . 18093526 10.1016/j.neuron.2007.10.037

[b40] Major G., Larkman A. U., Jonas P., Sakmann B. & Jack J. J. Detailed passive cable models of whole-cell recorded CA3 pyramidal neurons in rat hippocampal slices. J. Neurosci. 14, 4613–4638 (1994) . 8046439 10.1523/JNEUROSCI.14-08-04613.1994 PMC6577163

[b41] Lazarewicz M. T., Migliore M. & Ascoli G. A. A new bursting model of CA3 pyramidal cell physiology suggests multiple locations for spike initiation. Biosystems 67, 129–137 (2002) . 12459292 10.1016/s0303-2647(02)00071-0

[b42] Yang J. *et al.* Dopaminergic modulation of axonal potassium channels and action potential waveform in pyramidal neurons of prefrontal cortex. J. Physiol. 591, 3233–3251 (2013) . 23568892 10.1113/jphysiol.2013.251058 PMC3717225

[b43] Kopell N., Börgers C., Pervouchine D., Malerba P. & Tort A. in Hippocampal Microcircuits ed. C. V. Springer (2010) .

[b44] Tyzio R., Holmes G. L., Ben-Ari Y. & Khazipov R. Timing of the developmental switch in GABA(A) mediated signaling from excitation to inhibition in CA3 rat hippocampus using gramicidin perforated patch and extracellular recordings. Epilepsia 48, (Suppl 5): 96–105 (2007) . 17910587 10.1111/j.1528-1167.2007.01295.x

[b45] Pavlov I. *et al.* Tonic GABAA conductance bidirectionally controls interneuron firing pattern and synchronization in the CA3 hippocampal network. Proc. Natl Acad. Sci. USA 111, 504–509 (2014) . 24344272 10.1073/pnas.1308388110 PMC3890854

