# Supplementary material for: Presynaptic hyperpolarization induces a fast analogue modulation of spike-evoked transmission mediated by axonal sodium channels
Source: Nat Commun. 2015 Dec 10;6:10163. doi: 10.1038/ncomms10163 (PMC4682119; doi:10.1038/ncomms10163)
Supplement: Supplementary Information — Supplementary Figures 1-6 and Supplementary Table 1 [file ncomms10163-s1.pdf]

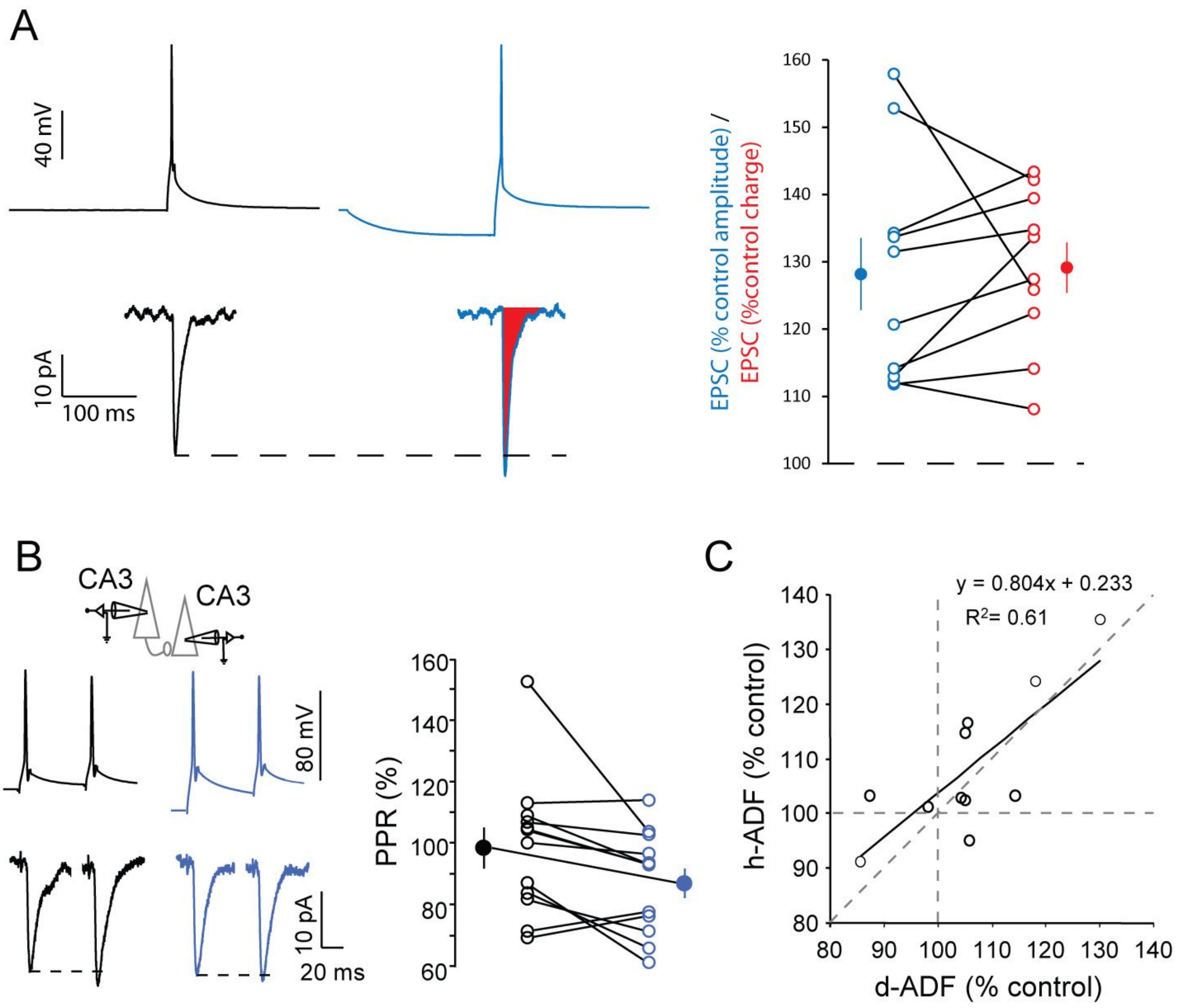

Supplementary Fig. 1. Properties of h-ADF in CA3 pyramidal neurons. A, Analysis of the EPSC charge. The potentiation is the same for amplitude and charge (128 ± 5 % and 129 ± 4 %, respectively, n = 10, Wilcoxon test, p>0.1). B, h-ADF is associated with a reduction of the paired-pulse ratio. Left, paired-pulse responses before (black) and after induction of h-ADF (blue). Right, quantitative data. Note the significant reduction in paired-pulse ratio (PPR) from control (black empty circles) to h-ADF (blue empty circles; n = 12, Wilcoxon, p = 0.021). C, Correlation between d- and h-ADF in the same CA3 neurons (n = 11).

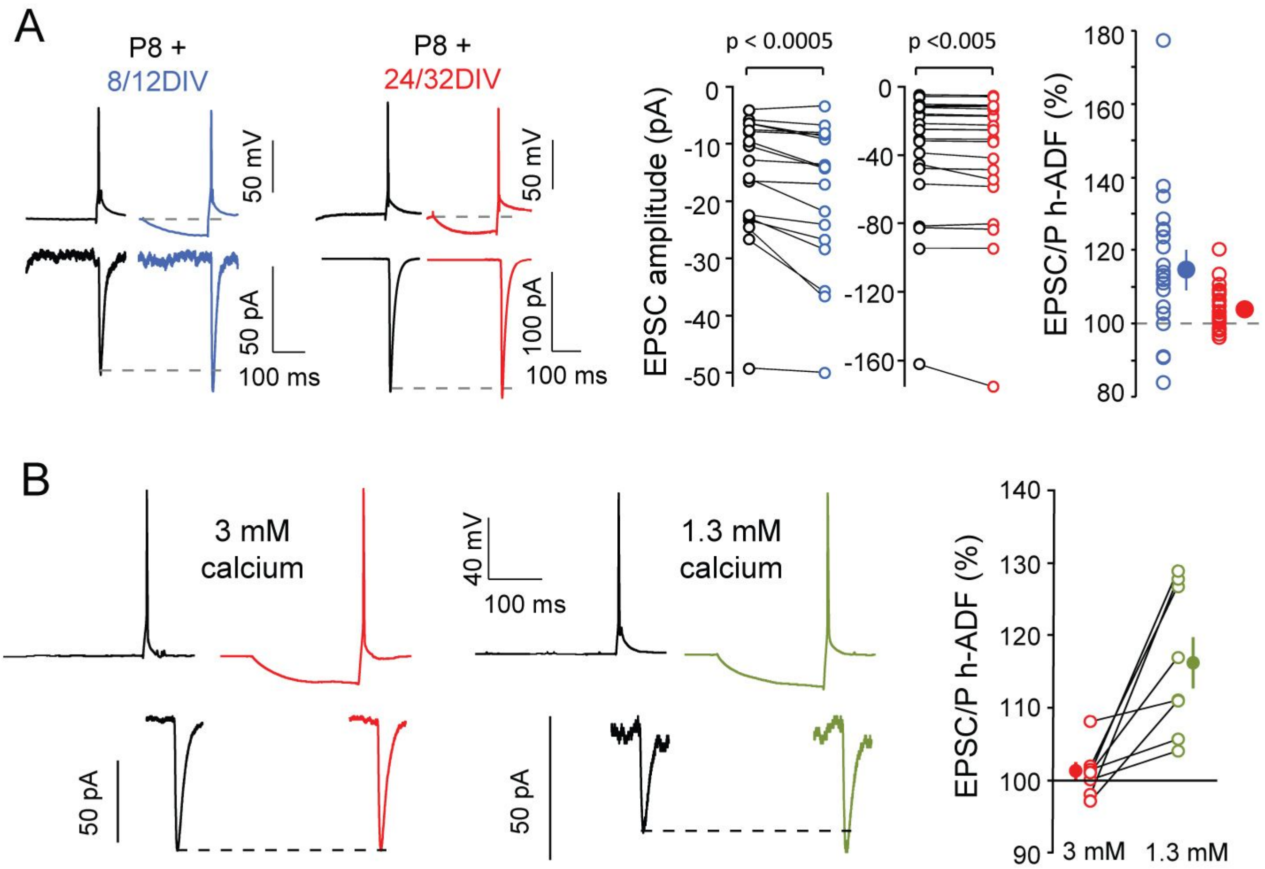

Supplementary Fig. 2. h-ADF is still present in mature cultures in physiological condition. A, Development of h-ADF. h-ADF is smaller in mature cultures (24/32 DIV) compared to young cultures (8-12 DIV). Left, representative examples. Middle, raw data for EPSCs recorded in 9/12 DIV (n = 17, blue circles; Wilcoxon test, p<0.0005) and in 24/32 DIV cultures (n = 20, red circles; Wilcoxon test, p<0.005). Right, group data of h-ADF (n = 21 for EPSCs/Ps recorded in 8/12 DIV cultures (blue circles) and n = 25 for EPSC/Ps recorded in 24/32 DIV cultures (red circles)).

B, h-ADF is restored in physiological calcium conditions. left, representative example. Right, quantitative data. Note the increase in EPSP/C amplitude when external calcium is decreased from 3 mM (red circles) to 1.3 mM (green circles, n = 8).

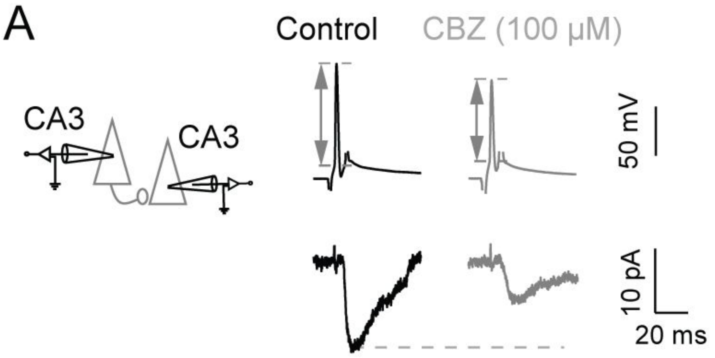

Supplementary Fig. 3. Effect of CBZ on synaptic strength and spike amplitude in CA3 neurons. A, Representative example of the effect of CBZ on synaptic transmission and spike amplitude. B. Quantification of the induced changes in action potential (AP, n = 10) and synaptic response (EPSP/C, n = 10).

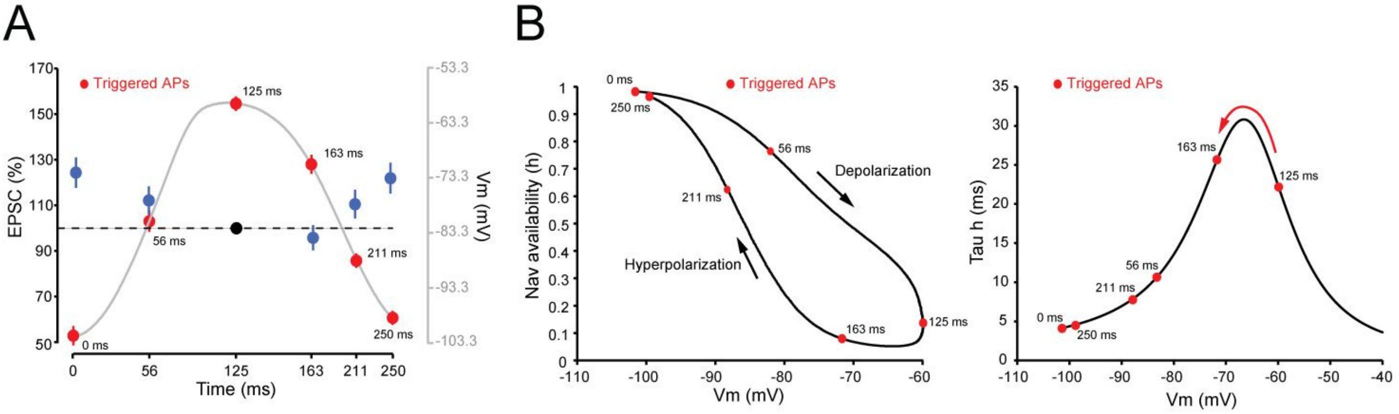

Supplementary Fig. 4. Effect of the oscillations on Nav kinetics. A, Simplified version of the EPSC modulation when injecting a 4Hz oscillation in the cell (modified Fig. 2). B, left, Nav channels availability in our Neuron model plotted in function of the potential during the oscillation. Note the quick inactivation upon depolarization and the slow recovery upon hyperpolarization. APs triggered at 125 & 163 ms are both in the really low availability area of Nav channels. Right, Tau h factor plotted in function to potential. APs triggered at 125 & 163 ms are both in slow domain of Nav kinetics (red arrow).

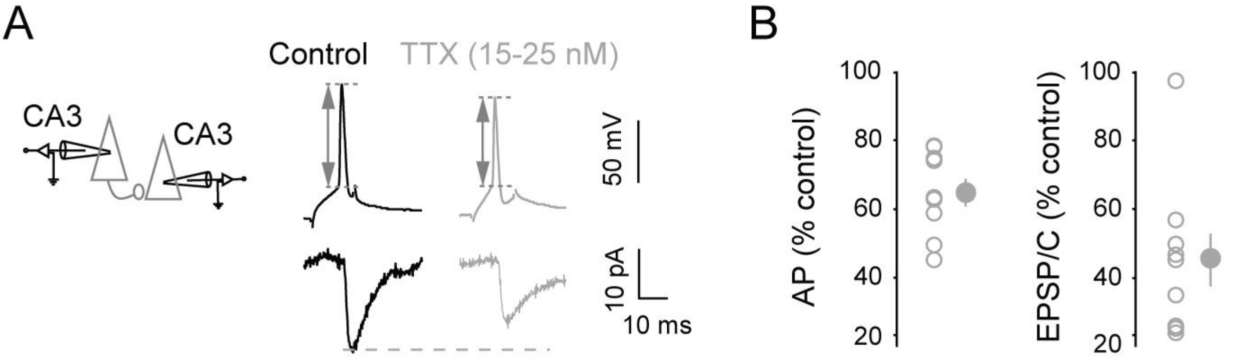

Supplementary Fig. 5. Effect of TTX on synaptic strength and spike amplitude in CA3 neurons. A, Representative example of the effect of TTX on synaptic transmission and spike amplitude. B. Quantification of the induced changes in action potential (AP, n = 9) and synaptic response (EPSP/C, n = 9).

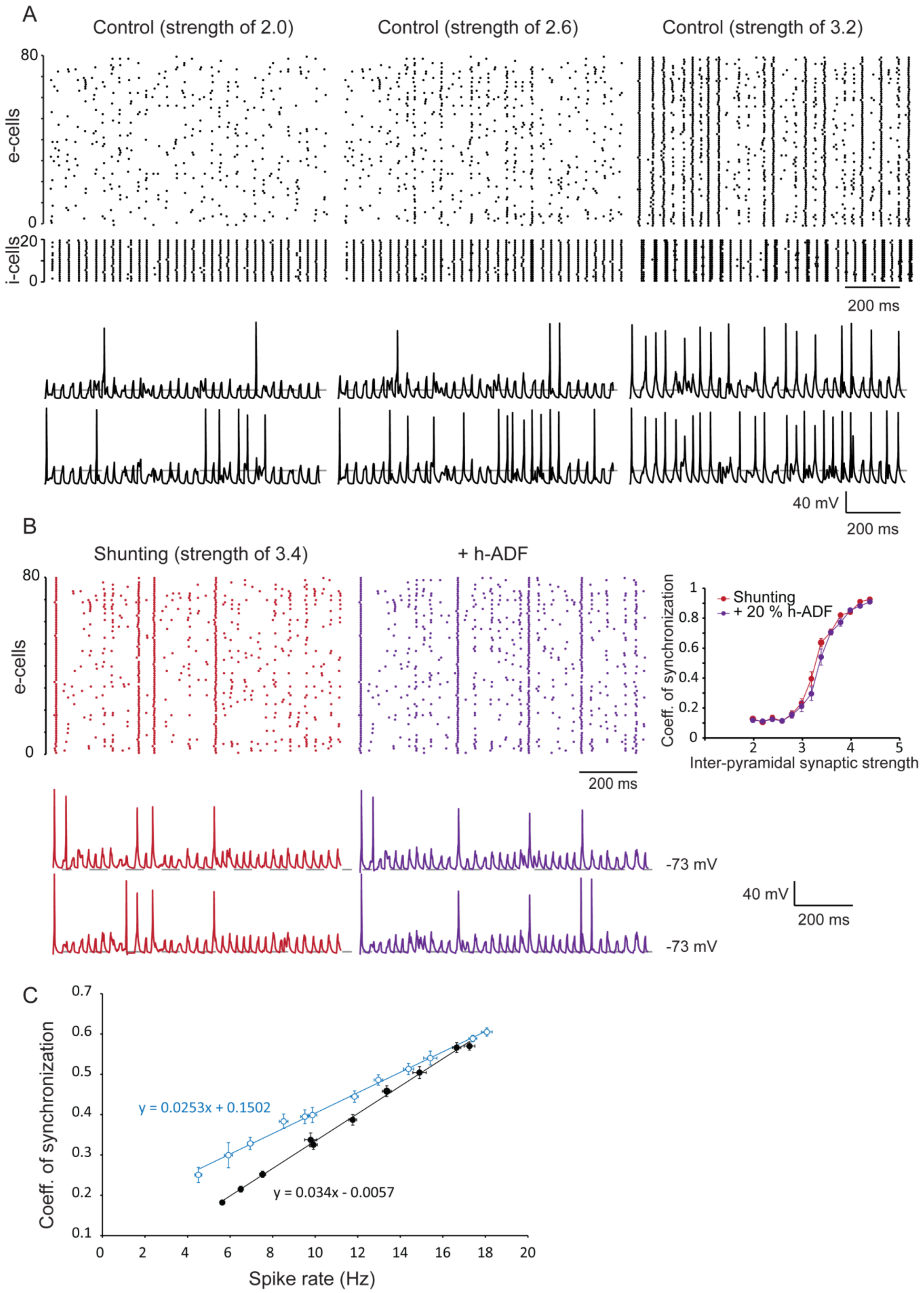

Supplementary Fig. 6 h-ADF and network synchrony. A, Rastergrams showing the progressive synchrony of the network when the synaptic strength is increased between pyramidal cells. With a

synaptic strength of 2.0 (left), there is no synchronization of the network. When the synaptic strength is progressively increased to 2.6 and 3.2 (middle and right), the network progressively synchronizes. e-cells: excitatory cells of the network; i-cells: inhibitory cells of the network. Bottom traces, activity in 2 e-cells in each case. B, h-ADF does not improve network synchrony with shunting inhibition (E_{GABA} = -65 mV). In this case, the strength between e-cells is 3.4 mS. Left and middle, rastergrams showing the lack of effect of h-ADF on network activity. Right, quantitative data (red, shunting (no h-ADF), violet, shunting + h-ADF). C, Role of spike rate on synchrony. Increasing spike rate enhances synchronization (black dots). In presence of h-ADF (blue dots), synchronization is further increased at any spike rate between 4 and 14 Hz (linear fit for each group of data, R^2>0.99 in each condition).

Supplementary Table 1

Model parameters

	Soma	Axon	Terminal
Length (µm)	30	200	2
Diameter (µm)	25	1	2
Membrane capacitance (µF/cm)	1	1	1
Passive conductance (S/cm^2)	2.6*10^{-4}	2.6*10^{-4}	2.6*10^{-4}
I_{Nasoma} (S/cm^2)	0.08	0	0
I_{Naaxon} (S/cm^2)	0	0.034	0.034
I_D (S/cm^2)	0	0.034	0.034
I_{KDR} (S/cm^2)	0.01	0.001	0.001
I_{Ca} (S/cm^2)	0	0	0.004
